# COVID‐19, nationalism, and the politics of crisis: A scholarly exchange

**DOI:** 10.1111/nana.12644

**Published:** 2020-07-19

**Authors:** Eric Taylor Woods, Robert Schertzer, Liah Greenfeld, Chris Hughes, Cynthia Miller‐Idriss

**Affiliations:** ^1^ University of East London London UK; ^2^ University of Toronto Ontario Canada; ^3^ Boston University Boston Massachusetts USA; ^4^ London School of Economics and Political Science London UK; ^5^ American University Washington, D.C. USA

**Keywords:** conflict, coronavirus, COVID‐19, crisis, nationalism, nation‐state

## Abstract

In this article, several scholars of nationalism discuss the potential for the COVID‐19 pandemic to impact the development of nationalism and world politics. To structure the discussion, the contributors respond to three questions: (1) how should we understand the relationship between nationalism and COVID‐19; (2) will COVID‐19 fuel ethnic and nationalist conflict; and (3) will COVID‐19 reinforce or erode the nation‐state in the long run? The contributors formulated their responses to these questions near to the outset of the pandemic, amid intense uncertainty. This made it acutely difficult, if not impossible, to make predictions. Nevertheless, it was felt that a historically and theoretically informed discussion would shed light on the types of political processes that could be triggered by the COVID‐19 pandemic. In doing so, the aim is to help orient researchers and policy‐makers as they grapple with what has rapidly become the most urgent issue of our times.

## 1 INTRODUCTION: ERIC TAYLOR WOODS (UNIVERSITY OF EAST LONDON) AND ROBERT SCHERTZER (UNIVERSITY OF TORONTO)

On March 11, 2020, Tedros Adhanom Ghebreyesus, the Director‐General of the World Health Organization (WHO), announced that COVID‐19 constituted a pandemic. It had become clear that it would not be possible to stop the worldwide spread of the virus and that it posed a global threat to health, economic well‐being and political stability. With regard to the latter, several leading political analysts suggested that the pandemic would fuel nationalism and usher in a more divided world (see Legrain, 2020; Allen et al. 2020). In response, the editors of *Nations and Nationalism* identified the need for an urgent discussion on how the pandemic might impact world politics, specifically in relation to nationalism. To that end, several scholars of nationalism, including Liah Greenfeld, Chris Hughes, Cynthia Miller‐Idriss, Robert Schertzer and Eric Taylor Woods, were invited to participate in this *Scholarly Exchange* on “COVID‐19, Nationalism, and the Politics of Crisis.”

Will the COVID‐19 pandemic lead to a rise in nationalist sentiment? How will the recent resurgence of nationalist populism across the globe shape the response to the pandemic? In this *Scholarly Exchange*, the contributors explore these themes to shed light on the relationship between nationalism and COVID‐19. The primary focus of the discussion is on whether COVID‐19 will fuel nationalism and ethnic conflict and how this will, in turn, impact the fortunes of the nation‐state. Of course, predictions are difficult, even in stable times. At the time of writing, we are still in the early days of the pandemic, when even the most basic terms of reference are difficult to grasp and changing fast. However, despite these challenges, we take the view that historically and theoretically informed reflection can be useful for identifying processes as they unfold. Ultimately, we hope that this will help researchers and policymakers as they respond to this urgent issue. It is in this spirit that this Scholarly Exchange was carried out.

Before getting into this discussion, it is important to clarify how we understand the COVID‐19 pandemic, from a political perspective. The pandemic is often described as a “crisis,” but additional specificity here can help us to map the potential political implications of COVID‐19. Crises are triggered by real or perceived threats. They are highly disruptive events (upending plans, routines, expectations, beliefs and values) that create heightened uncertainty (Brecher, 2019; Quarantelli & Dynes, 1977; Rosenthal, Charles, & Hart, 1989). This disruption and uncertainty can shift the *context* within which politics occurs (Falleti & Lynch, 2009). These types of shifts—often conceptualized as “critical junctures”—tend to be triggered by external shocks that lead to structural indeterminacy (Falleti & Lynch, 2009; Mahoney 2001; Capoccia & Kelemen, 2007). COVID‐19 fits all these criteria. The threat posed by the pandemic has already triggered a series of cascading crises in health and economics, and it now threatens to overwhelm politics and upend the nationalist context in which much of world politics occurs.

What, then, is nationalism? When expressed as a political ideology, nationalism holds that territorial communities called nations are necessary for human flourishing and that each nation should therefore be accorded a degree of autonomy in determining its own affairs. This idea provides the rationale for self‐determination in world politics. Communities that can legitimately claim that they are a ‘nation’ should, in theory, have a right to self‐determination. But nationalism is more than a political ideology; it is also an important source of meaning and identity. As such, it provides a framework for unity and division. At its most powerful, nationalism can inspire such strength of feeling that people are willing to die for their fellow nationals. By the same token, it can arouse deep‐seated hatred and violence against foreign others. Despite frequent predictions to the contrary, nationalism remains central to world politics. Indeed, COVID‐19 arrived at a time when nationalism was undergoing a resurgence (Bieber 2018; Halikiopoulou & Vlandas 2019). In many cases, this “new nationalism” has taken on a decidedly ethnic and populist dimension (Bonikowski, 2017; Schertzer & Woods, 2020b).

To reflect on the potential impacts of COVID‐19 on nationalism and the nation‐state system, the contributors to this *Scholarly Exchange* have been asked to address three related questions: (1) how should we understand the relationship between nationalism and COVID‐19; (2) will COVID‐19 fuel ethnic and nationalist conflict; and (3) will COVID‐19 reinforce or erode the nation‐state in the long run? Together, these questions allow the contributors to reflect on how COVID‐19 may affect nationalism and the nation‐state and how these core aspects of politics will in turn shape the response to COVID‐19.

The first question asks contributors to explain how they understand nationalism, how it may shape the response to COVID‐19 and whether COVID‐19 will in turn impact on nationalism. In their replies, Greenfeld, Hughes and Miller‐Idriss discuss the ways in which nationalism is shaping the response to the pandemic. Greenfeld argues that ethnic nationalism is a key variable shaping the responses of many states to COVID‐19 when compared with previous pandemics such as H1N1. Miller‐Idriss strongly agrees and points out that states led by populist nationalists are faring much worse than others. Hughes picks up on these themes by arguing that the medical and health care response is being 'weaponized' to support nationalist aims. On the other hand, the contributors argue that COVID‐19 will also shape nationalism. On this point, Woods and Schertzer put forward a typology for analysing how the pandemic could affect the development of nationalism, arguing that it could be constitutive, amplifying or transformative. Ultimately, among these three possible trajectories, they argue that the most likely impact of the pandemic will be to amplify existing ethnic and national cleavages. Miller‐Idriss joins Woods and Schertzer in highlighting this amplifying effect by pointing to rising anti‐immigrant, xenophobic and conspiratorial anti‐state sentiments in many states in the wake of COVID‐19. Hughes takes a slightly different view here by drawing attention to the potential for COVID‐19 to act as a transformative moment in Chinese nationalism that revolves around pride for containing the virus.

The second question asks contributors to reflect upon one of the most pressing issues on people's minds today—the potential for COVID‐19 to trigger and enflame ethnic and national conflict. Several commentators have already raised the possibility of large‐scale global warfare, given parallels to the decades following the Spanish flu in 1918 and the economic ruin of the interwar period. However, there are many different types and levels of conflict: ethnic and national conflict has external (interstate) and internal (intrastate) dimensions, and it runs the gamut from largely peaceful political conflict to outright violence and warfare (Schertzer & Woods, 2011). In their responses, the contributors consider these differing dimensions of conflict. Focusing on the potential for COVID‐19 to exacerbate conflict between China, Hong Kong and Taiwan, Hughes notes how the pandemic has already provided the Chinese government with a pretext for accomplishing its nationalist aims of integrating the territories with the mainland. On the other hand, Hughes observes that Taiwan has also used COVID‐19 to secure greater visibility in the international arena. This, in turn, risks drawing in more state actors, to become a larger interstate conflict between China and the West, particularly with the United States. On this score, Greenfeld agrees that COVID‐19 risks amplifying conflict between China and the United States. Greenfeld also comments on the possibility of the pandemic amplifying ethnic nationalism leading to the persecution of ethnic minorities. Miller‐Idriss similarly focuses on the potential for COVID‐19 to increase persecution of minorities, noting the rise in anti‐Asian racism in the United States. However, for Miller‐Idriss, it is in the fragile states of the global south where the risks of nationalist and ethnic conflict are greatest. For their part, Woods and Schertzer discuss many of these same themes, highlighting the specific risks that occur when a “politics of blame” is combined with nationalism. They argue that this combination can increase the risks of conflict with individuals and communities who are perceived as 'others.'

Finally, the third question asks contributors to project forward and reflect on whether the pandemic will have a lasting impact on the building block of our international order—the nation‐state. As noted above, COVID‐19 arrived in a world where nationalism, trade protectionism and migration controls were on the ascent. It is possible that the pandemic will amplify these forces, leading nation‐states to turn further inward. At the same time, the global nature of the virus may force international collaboration to mount an effective response. Among these possible futures, no matter how much upheaval that may be caused by COVID‐19, Greenfeld doubts that it will shake the ideal of the nation‐state as a vehicle for securing the identity and dignity of its citizens. Quite the opposite, Greenfeld suggests that the pandemic will work to erode global institutions. Here, Hughes broadly agrees with Greenfeld, while also highlighting the ways in which the response to the pandemic could be used as a cover to strengthen the nation‐state. Hughes also warns that even the scientific community may not be immune to a process of nationalization. Miller‐Idriss parts ways from Greenfeld and Hughes by suggesting that while the powerful nation‐states may be strengthened by COVID‐19, it is likely that the pandemic will erode the more fragile states of the global south. Woods and Schertzer pick up on this theme, while also pointing to a range of potential threats that COVID‐19 may throw at the nation‐state, whether “from above” by neo‐imperialisms or “from below” by new nationalist movements. However, while these threats may undermine some individual nation‐states, they argue, like Greenfeld, that this will not necessarily erode the potency of the nation‐state as an idea.

## 2 LIAH GREENFELD (BOSTON UNIVERSITY)

To answer these questions, as any question regarding the relationship between nationalism and other phenomena, it is necessary, first, to have a clear understanding of how cultures and societies function and evolve, in general, and of the nature of the cultural and social phenomenon of nationalism, specifically. To address these issues in 2,000 or so words fully is impossible, so I shall only state the empirical conclusions of my investigations and proceed on this basis. Even these introductory remarks, however, must be introduced with a methodological consideration. When I am talking about a clear understanding, I do not mean to say “my understanding”; this would be tantamount to a chemist, for instance, saying “my understanding of gas is such and such,” presuming that someone else's understanding is expected to be different. Rather, assuming that cultures, societies and nationalism, just like gases, are empirical phenomena, I am approaching social and historical facts, following classical methodological recommendations, as things, without any prejudgement (Bloch, 1964; Durkheim, 2014).

The cultural (social, political, economic, etc.) process occurs simultaneously on the level of the individual mind and the collective level of the surrounding culture and consists of the constant give and take between these two levels. Every collective trend begins with a new individual experience, to which the mind will react using existing cultural resources, but the reaction may be creative, that is, unpredictable. If the experience is sufficiently provocative and common, a new interest may transform this creative individual reaction into a shared ideal, eventually resulting in a new way of thinking and acting, that is, give rise to a new social institution, adding to the cultural resources and changing the institutional structure—the nature—of a society (Greenfeld, 2013, 2016, 2019). This, in most basic terms, is how societies change and evolve, in general, and how nationalism evolved, in particular.

In regard to nationalism, specifically, one must keep in mind the following. (a) It is a historical, modern phenomenon: before the 16th century, there were no nations. (b) It is essentially a way of thinking, the basis of any institutional structure—thinking that the social world is naturally divided into sovereign communities of fundamentally equal members (communities called nations) and that a just society, consistent with the human nature, therefore, is an egalitarian society based on the principles of popular sovereignty. (c) As a result, nationalism implies democracy, every nation being a democratic society by definition. (d) The democratic/national principles of fundamental equality of membership and popular sovereignty can be interpreted and implemented differently, producing several types of nationalism: not every nationalism is ethnic. In the monotheistic civilization alone, in which ethnic nationalism is indeed the most common type, there exist two other types of nationalism: individualistic nationalism (such as the original English one) and collectivistic‐civic nationalism (such as the French). (e) The broad appeal of nationalism is due to the fact that fundamental equality of membership in the nation and the consciousness of popular sovereignty dignify personal identities of members of the nation (Greenfeld, 1992).

### 2.1 How should we understand the relationship between nationalism and COVID‐19?

In this framework, we can examine the relationship between nationalism and COVID‐19. Let us begin with the possible effects of nationalism on the course of the pandemic. The course of the pandemic was certainly affected by the ways it was handled in different countries, and the way it was handled, I would argue, was a direct function of nationalism, specifically, of the national conflict between China and the United States.

What leads me to say this? The comparison between coronavirus and previous pandemics: be it H1N1, SARS, MERS, Ebola, HIV‐AIDS of the recent decades, or such historically remote lethal attacks of infectious disease as the Spanish flu of the last century or the plague (Black Death) in the Middle Ages. None of the previous pandemics involved worldwide lockdowns, cessation of normal activities and massive state‐sponsored and state‐controlled mitigation. Both the Black Death, which, incidentally, also came from China, and the Spanish flu were incomparably more lethal than coronavirus: the plague would kill one in two people, 50%, in settlements it reached; if one contacted it, the fatality rate was between 85% and 100%. Yet, only a few governments, such as that of the city of Milan, ruled by a most brutal dictator, attempted to mitigate (Benedictow, 2004). Of course, one can argue, no government at the time but a brutal dictatorship in a small city‐state had the means to control its population (and the spread of the disease) to the extent that nation‐states of today have. But this cannot be said of the influenza of 1918 and even less of recent pandemics (SARS, H1N1, etc.), when the means of disease control at the disposal of governments were identical to what they are now. SARS and H1N1, for instance, were at least as frightening as coronavirus (Snowden, 2019). But no worldwide panic ensued. Neither the world economy nor that of any separate nation came to a standstill. One may argue that all of the recent pandemics proved far less devastating than was originally expected. But mitigation of coronavirus on a massive, coordinated scale began *before* it was known how devastating it might be (which is still not really known): reports that China was investigating a respiratory illness in Wuhan appeared only on December 31, 2019. The Chinese government imposed a lockdown on the 10‐million‐large city of Wuhan on January 23, 2020. On January 29, when only one case of infection on the American soil was identified, the coronavirus task force was created in the United States, and on January 31, a ban on travel from China was imposed. Closure of inessential businesses and schools, stay‐at‐home orders and construction of new medical coronavirus‐ready facilities in record‐breaking times followed. Although the response of some other countries (Japan, South Korea and Taiwan in the immediate vicinity of China, but also Italy) was independent, most of the world was directly influenced by the reaction of China and the United States. Within the first two months of the pandemic, economies contracted around the world, registering negative growth, unemployment skyrocketed, lives were universally disrupted, leaders interpreting this as they would results of a war on domestic soil and publics taking this in stride as they would indeed a war effort.

What was different in the cases of H1N1, for instance, and coronavirus in the first two months of the declared pandemic? Nothing. The difference in the reaction was not a function of the known difference in the nature or threat of the virus; it was a function of a difference in the political configuration of the world at the two points in time when the virus appeared. In April 2009, when H1N1 was first reported, the United States was still the one uncontested (though resented and attacked) superpower in the world. No nation yet took the place of the Soviet Union vis‐à‐vis it, challenging its position as the world's leader and arbiter. The superiority, the dignity and authority of the United States were beyond competition, if not beyond idle question. In 2020, this was definitely not so. China, which only announced its nationalism in 2008 at its coming out party during the Beijing Olympics, has been steadily and at an increasing speed gaining on the United States in this competition in the past 11 years, and the chief American national interest—the interest in superiority, dignity and authority—was now at stake. Chinese leadership used coronavirus (whether intentionally or not) to challenge the United States to a single combat, so to speak. Could you match us, President Xi essentially offered, in containing a pandemic? The United States could no more disregard this challenge than it could disregard the Sputnik in 1957. And so, the public health race started: who could build a larger hospital in a shorter period of time, produce more PPE, administer more tests, stop outbreaks sooner and ensure more cooperation from the population? What could the rest of the world do, but follow the example of the two giants, disputing who would preside over it past 2020?

### 2.2 Will COVID‐19 fuel ethnic and nationalist conflict?

Now let us address the question of the possible effects of the pandemic on nationalism. Would it, for instance, strengthen nationalist and especially ethnic‐nationalist conflicts? Given the news reaching us from Washington and Beijing, it seems clear that it has strengthened the nationalist conflict between the United States and China, which is as momentous as a nationalist conflict for the world today, as the nationalist conflict between the United States and Russia (ruling over the Soviet Union) was at the time of the Cold War. It is also quite clear that the pandemic had brought to the surface the nationalist conflicts within the EU, undermining the confidence in globalization in the one region which has been seen by experts as its empirical proof—the proof that human society was becoming transnational, transcending its national stage and moving towards a global community—and even among its staunchest erstwhile supporters. The universal reversion to nationalist policies and defence of particularistic national interests at the expense of transnational solidarity during the pandemic, however, only proved that rumours of nationalism's demise in the core Western European nations have been grossly exaggerated. Widespread manifestations of Euroscepticism, such as strong national feeling in France, Italy, the Netherlands and so on, or even Brexit, have not been regarded by theorists of globalization as an empirical contradiction of their theoretical position, but as proof of reactionary, right‐wing or even extreme right political agenda of populist leaders and benighted, false consciousness among their uneducated followers. Now it is obvious to all that the theorists were wrong (though how long this would remain obvious is another question): thanks to the pandemic, globalization today no longer seems the obvious current stage of human development, and nationalism no longer appears as the stage obviously transcended. Nationalism in Western Europe (in distinction to Central and Eastern Europe, for instance) has traditionally not been ethnic however, but rather individualistic, as in Britain, or collectivistic‐civic, as in France, Italy and Spain.

Would the pandemic fuel ethnic nationalist conflicts? The psychological foundation of ethnic nationalism is *ressentiment*, that is, existential envy, which is most efficiently assuaged by the humiliation to the point of elimination of the envied other; therefore, ethnic nationalism is inherently aggressive (Greenfeld, 1992; Greenfeld & Chirot, 1994). Where it exists, anything can serve as fuel for ethnic aggression. The pandemic has already added to anti‐Semitic conspiracy theories (very much in line with medieval poisoning of the wells narrative born during the Black Death) in Palestine and among certain publics in Europe (ADL, 2020). Anti‐Semitism, of course, is the most deeply embedded institution (established way of thinking and acting) in the monotheistic world, predating nationalism by many centuries—and for this reason offering a particularly virulent and reliable channel of expression to ethnic nationalism—but one can imagine temporary flare‐ups of less widespread ethnonational hostilities, in which a group identifies the object of ethnic national antagonism as the carrier of the virus.

### 2.3 Will COVID‐19 reinforce or erode the nation‐state in the long run?

And, finally, will COVID‐19 reinforce or erode nation‐state—that is, nationalism, nationalist institutions—in the long run? Leaving aside the question of what can be said about the long run, in general, we should consider this in the wider framework of processes involved in social change, briefly sketched above. To use the most striking example of the social disruption caused by a sudden assault of infectious disease, the Black Death, the plague might have disrupted the medieval society of orders and shaken this social structure. Land became cheap and labour dear, which allowed people from the lower classes to behave as if they belonged to the upper ones and encouraged intermarriage between poor noblemen and daughters of rich commoners. However, as with a dilapidating building, the unravelling of society did not in itself provide any orientation for reconstruction. The thinking remained the same, and for several centuries after the plague years of 1348–1350, the reconstruction took the form of piecemeal patch‐ups: sumptuary laws characterized the period, reflecting both that the old order was unravelling and that the only way society was imagined was exactly as it had been before the pandemic (Cantor, 2001; Cohn, 2010; Herlihy, 1997). Only when reality was *reimagined* and new (national, as it happened) consciousness appeared did the direction of reconstruction became clear and set. Institutions, which, as already Durkheim emphasized, are just established ways of thinking and acting, are never stable—they are always in the process of waxing and waning, strengthening, weakening and modifying. Conceptualization—ways of thinking, that is, to use Weber's terminology, *ideals*, especially if encoded in laws, sacred texts, whether religious or secular, such as the American Declaration of Independence, popular and high culture, and so on—is always their strongest feature, but can rapidly be abandoned, if the *interests* supporting these ideals disappear. The interest behind nationalism and its institutions (e.g., nation‐state)—dignity of personal identity—is alive and well. COVID‐19 also ranged behind it the essential material interests (health, life and livelihood), pointing at the same time to the inability of transnational institutions—globalization—to serve these interests. It is transnational institutions, rather than nation‐state, that are likely to fall victim to the pandemic.

## 3 CHRISTOPHER HUGHES (LONDON SCHOOL OF ECONOMICS)

I would like to interpret the three questions as addressing national identity, policy‐making and state‐building, respectively.

### 3.1 How should we understand the relationship between nationalism and COVID‐19?

Greenfeld is right to highlight how COVID‐19 has been politicized by growing tensions between the United States and China over a range of issues. This can be explored further by looking at the way in which COVID‐19 is being used in a process of mutual identity construction, which makes it impossible for medicine and science to be politically neutral. A good starting point is the naming of the virus. WHO has been aware that identifying the geographical origin of a virus can provoke a backlash against members of a particular religious or ethnic community since 2015, at least, when it produced guidelines that call on governments to avoid this (WHO, 2015). The convention was breached when the Trump administration saw political mileage in using labels such as “Wuhan virus” and “China virus” instead of the neutral name “COVID‐19,” leading the Chinese government to castigate it as suffering from an “ideological virus” (People's Daily, 2020; Wang, 2020). This is despite the fact that the Chinese government had already used the name “Wuhan virus” to imply that the epidemic was a localized outbreak.

It may be true, as Greenfeld points out, that there has been an unprecedented scale of international coordination to contain COVID‐19, but the history of pandemics shows that measures to control movement can be used to form national identity. This actually goes back to the age of empire, when thousands of Muslim pilgrims were detained under sanitary controls imposed on the Red Sea area. In contrast, few people called for the quarantine of Lawrence of Arabia or other allied soldiers returning from the Middle East in World War I (Chase‐Levenson, 2020). A similar dynamic can be seen when various countries responded to COVID‐19 by imposing bans on travel from China in February 2020, motivating the Chinese government and commentators to make accusations of racial prejudice. Beijing's ambassador to Israel even went so far as to liken the closure of borders to the turning away of Jewish refugees during the Holocaust, which the embassy had to quickly retract (The Guardian*,* 2020).

This is typical of the process by which governing elites use the spectre of the external enemy, or the “other,” to build national identity, that Woods and Schertzer draw attention to. At present, China and the United states are clearly using COVID‐19 in this way. This is illustrated by the controversy that blew up when the *Wall Street Journal* published an article titled “China is the Real Sick Man of Asia” in February 2020 (Mead, 2020). China reacted by expelling three of the newspaper's reporters, while its Foreign Ministry warned that it “must be held responsible for what it has said and done,” to which US Secretary of State Mike Pompeo responded that “mature, responsible countries understand that a free press reports facts and expresses opinions.”

It is also important acknowledge that the pandemic is being used for a more positive construction of identity. This is quite clear in the way that the Chinese government is using its apparently successful containment of the pandemic to propagate the superiority of the “China Model” of politics, after the legitimacy of the Chinese Communist Party (CCP) was badly dented by the early mismanagement of the crisis. By describing the campaign in terms of a “people's war,” it can also be linked with the narrative of the CCP's “salvation of the nation” from Japanese aggression and misrule by the Nationalists in the 1930s and 1940s.

### 3.2 Will COVID‐19 fuel ethnic and nationalist conflict?

One of the most disturbing aspects of the COVID‐19 crisis is the way in which it is used to weaponize medicine in ethnic and nationalist conflicts. This is most evident in the CCP's attempts to exert control over territories that are central to its nationalist mission. It could already be seen in the summer of 2019. During that time, medics were subjected to police intimidation, arrest and surveillance as they came to the aid of citizens who were injured in demonstrations against the introduction of a law to extradite residents to mainland China, according to Dr Darren Mann's eyewitness testimony before the House of Lords on December 18, 2019. Such behaviour is in breach of the principle that access to treatment is a universal right without distinction of race, religion, political belief and economic or social condition, as enshrined in the WHO charter. This was already a strong deterrent to demonstrators when Beijing took advantage of a ban on mass gatherings in the territory to impose a National Security Law in May 2020, which will criminalize criticism of the CCP as unpatriotic and secessionist. When demonstrators defied the ban, they were condemned as a “political virus” (SCMP, 2020).

The use of COVID‐19 to fuel a nationalist conflict can also be observed in China's insistence that Taiwan should be excluded from WHO, on the grounds that it is a part of China, despite its excellent record in containing the pandemic. It is too early to know how COVID‐19 will shape Taiwan's identity politics, but the same situation during the SARS epidemic of 2002–2003 allowed its incumbent president to use the “Chinese plague” to galvanize flagging support in the polls by holding a referendum on demanding representation in international organizations, which helped him to win re‐election in 2004. Given that an opinion poll conducted before the virus hit Taiwan shows that the proportion of the population who self‐identify as Taiwanese has already risen to a new high of 66%, while 28% identify as both Taiwanese and Chinese (Pew, 2020), Taiwan's current president can gain substantial political capital by ramping up the campaign for WHO representation.

The potential for COVID‐19 to fuel a nationalist conflict is further heightened when such issues become part of global and regional geopolitics. This is deepening as Taiwan gains substantial support from other democratic states, while China appears to be taking advantage of the health crisis to step up its naval and air force intrusions into the waters around the island and into the South China Sea.

This growing linkage of the pandemic with the national security of the United States and China creates a context within which individuals in both countries are likely to be harassed as carriers of COVID‐19, especially in the context of the rising populism that is highlighted by Miller‐Idriss. This can be seen in the United States, where anybody deemed to be “Chinese” due to their East Asian features has become more liable to be harassed and assaulted. In China, where popular nationalism has long been used by the CCP as a source of legitimacy, xenophobia been fed by the narrative that the Party is fighting and winning a “war” against a virus that was sent by the United States and is being spread by foreigners. There have been particularly serious cases of racism towards Africans, due to the erroneous belief that they are unhygienic carriers.

### 3.3 Will COVID‐19 reinforce or erode the nation‐state in the long run?

While Miller‐Idriss is right to point to the ways in which COVID‐19 has been used to fuel anti‐government extremism and conspiratorial sedition, it is also possible to find examples where civil society actors have criticized its use for nationalistic purposes: some 53 reporters and editors at the *Wall Street Journal* signed a letter calling for the “sick man” headline to be changed and for an apology to be made; Chinese commentators have pointed out that it was their own intellectuals who began to refer to their country as a “sick man,” going as far back as the defeat of the Qing Empire by Japan in 1895. Such voices will remain marginal compared with the advocates of nationalism, however, unless COVID‐19 gives medical science sufficient authority to force the kind of cooperation between states that will weaken national sovereignty.

History provides little evidence to support this prospect, however. From the coordination of quarantine procedures between the Italian city‐states down to today's WHO, contagious diseases have allowed governments to steadily accrue power over their citizens. China's use of information and communications technology to surveil its citizens as it manages COVID‐19 marks a new stage in this process. The dangers posed to civil liberties in democratic systems are also shown by cases such as the use of mobile telephones to identify and trace a disproportionate number of South Korea's LGBT community, who face serious discrimination as a result. The state will become even more powerful if COVID‐19 justifies the introduction of new barriers to migration and the targeting of border health checks according to the national origins of travellers.

The current crisis also shows how disease can be used not only to undermine the authority of scientists and medics, as Miller‐Idriss points out, but also to turn them into political actors and national symbols. Most controversial is the casting of Dr Li Wenliang, as a “martyr” after he died from the virus, despite having been detained and disciplined by the authorities for trying to warn his colleagues at the early stage of the outbreak in Wuhan. He was also posthumously awarded the May 4 Youth Medal, a name that refers to the student demonstrations against the transfer of German concessions in China to Japan at Versailles, in 1919, seen by many as the birth of Chinese mass nationalism.

The judgement of scientists according to their political loyalties is also being extended to international organizations. Most notable is the accusation by the United States that the director of WHO, Dr Tedros Adhanom Ghebreyesus, is an agent of China. Again, this is not new. The Jewish‐Polish epidemiologist, Ludwik Rajchman, who took the leading role in building the precursor of the international health system of the League of Nations, might well have been appointed the first director of WHO, had he not worked on epidemics in China in the 1930s. Instead, he became a victim of the purge of Communist sympathizers in the United States after World War II, even though he had worked most closely with the ruling Nationalists in China (Belińska, 1998).

The harnessing of scientists and medics to the nation‐state can even be traced back to the China's first international conference, a meeting of epidemiologists in 1911 to discuss a pneumonic plague that had killed some 60,000 people in Manchuria. To be sure that a “Chinese” scientist should play a leading role, the Qing Empire appointed Dr Wu Lien‐Teh (1879–1960) as its representative, despite the fact that he was born in Malaysia and was thus a subject of the British empire. Having been on the receiving end of the racism of European scientists and diplomats, Wu was happy to lead a project that was partly seen as a way to prevent Japan and Russia from using the plague to assert their growing control over Manchuria (Wu, 2004). He would go on to become an authority in the emerging international health system, challenge British interests in Malaya by establishing an Anti‐Opium Society, campaign to remove racial discrimination in the provision of public services, and co‐author a history of Chinese medicine (Wu & Wong, 1977).

Members of the scientific community can thus be agents of nationalism as much as they can be a force for cooperation. The latter becomes less likely as the decoupling of the United States and China requires them to prove their loyalty or face accusations of subterfuge or even espionage. The need for states to ensure self‐sufficiency and reliable partnerships for the supply of essential medicines and protective equipment is also leading to the securitization of health, which will accelerate the deglobalization of trade and the movement of people. The recent decision of the United States to withdraw from WHO due to its handling of the pandemic thus looks more like a throwback to the years when sovereignty trumped international cooperation and brought down the League of Nations than a world in which the nation‐state is in decline. I thus agree with Greenfeld that transnational institutions are more likely than the nation‐state to be damaged by the pandemic.

## 4 CYNTHIA MILLER‐IDRISS (AMERICAN UNIVERSITY)

There are several dimensions to the relationship between nationalism and COVID‐19 that ought to be disentangled, but first, let me be clear about how I understand the concept of nationalism itself and the version of it I analyse here. Nationalism is an exclusionary political project to make the state congruent with the nation (Fox and Miller‐Idriss 2008). This can take many forms, from fully secessionist and independence movements to xenophobic and anti‐immigrant expressions within an existing state. The current form of nationalist governance that we have seen emerge in several global states is what I call populist nationalism. Populism is both a schema (way of thinking) and a rhetorical strategy that pits the ordinary, pure people against the corrupt elites (Bonikowski, 2017; Canovan, 1999; Brubaker, 2017; Miller‐Idriss 2019; Mudde, 2004; Müller, 2016).

Populist nationalism, in turn, extends this pure people‐nefarious elite dichotomy to a framing in which all “others” pose an essential threat to the pure nation and its ordinary people. Only a stronger state, so the argument goes, can protect the nation from the growing danger posed by immigrants, ethnic others, non‐Christian religions and more. This is what Jan Kubik (2018) calls a “thick” form of populism, in contrast to Mudde's (2004) description of populism as having a thin ideology. Others have described “thick” populism using slightly different terms, such as Rogers Brubaker's classification of vertical and horizontal dimensions of populism, where the vertical dimension positions the people against the elite and the horizontal dimension creates intense polarization and fixed boundaries between groups of people (Brubaker, 2017; Berezin, 2019; Kubik, 2018; Miller‐Idriss, 2019). Populist nationalism is the form and expression of nationalism that I refer to in this essay, although I will also use the shorthand “nationalism” to refer to it.

### 4.1 How should we understand the relationship between nationalism and COVID‐19?

With this understanding of nationalism, I return to the relationship between nationalism and COVID‐19. I suggest that there are at least three major impacts to explore. First, early indicators suggest that there is a direct impact of populist nationalism on the public health, infection rates and mortality rates of COVID‐19. As I write this, several of the countries in the world with the highest COVID‐19 infection rates are led by populist nationalist leaders—including the United States, Brazil and the United Kingdom. The United States alone is responsible for over a quarter each of COVID‐19 infections and deaths globally, although the US population represents only 4.25% of the global population (see World Health Organization, 2020, online; United States Census Bureau, 2020, online). Why would populist nationalism itself be detrimental to a public health crisis? One reason is that populist nationalists' attacks on the “corrupt elite” have gone well beyond critiques of political leaders and opponents to include other “elite” experts, academics and scientists, as evidenced by a rejection of climate science and global environmental agreements, for example. Undermining and delegitimizing scientific expertise and global cooperation and information sharing makes it significantly more difficult to convince the public of the benefits of shelter in place orders or practices to reduce the spread of the disease. In the case of COVID‐19, populist nationalist leaders are thus more likely than other national leaders to reject scientists' advice, attack global organizations like WHO, promote scientifically unproven and potentially harmful treatments for COVID‐19 and reject scientifically proven practices like wearing masks in public. Populist nationalist anti‐elite and anti‐science sentiments have undoubtedly led to higher COVID‐19 infection and mortality rates as a result.

Populist nationalists do not only attack and undermine scientific expertise, of course. The purity of the people, within populist nationalist frames, rests both in contrast to corrupt elites and to racial, ethnic, religious and immigrant “others.” This is where the second impact of nationalism on COVID‐19 outcomes becomes clear. Across Europe and North America, there has already been a documented rise in xenophobic, anti‐immigrant, anti‐Asian and anti‐Semitic hate during the global pandemic. The US administration's insistence on using the term “Wuhan virus” or “Chinese virus” is one of “many strategies of apportioning the blame for the (spread of the) virus to a specific place/country and to construct the disease as a foreign‐grown threat to the nation” (Nossem 2020: 5). In the United States alone, over 1,700 anti‐Asian hate incidents were reported within the first 6 weeks of a new website established by Asian American and Pacific Islander civil rights groups (Lee & Yadav, 2020), to name just one example (see Stop AAPI Hate Reporting Center, n.d. online).

Such xenophobic expressions of nationalism are part of a clearly documented, pre‐COVID‐19 rise in far right and extremist hate and the legitimation of White supremacist extremism (Ebner, 2020; Miller‐Idriss, 2018, 2020; Mudde 2019). White identity and the need for its protection and defence is a common thread across White supremacist and White nationalist beliefs and practices (Belew, 2016). During the COVID‐19 pandemic, these expressions have found a home in the circulation of memes and social media commentary that scapegoat entire populations as being responsible for the virus and its spread (see Anti‐Defamation League, 2020). Dehumanizing language about “dirty” immigrants carrying disease has accompanied immigration bans along with border closures, asylum application denials, deportations and more, even while the practices of local “native” populations that rapidly spread the virus in local churches, parties, funerals, ski lodges and more have continued in more or less unchecked ways. At the extreme fringe, moreover, there are clear risks that the COVID‐19 era will help reinforce White supremacist extremists' sense of White victimhood and concomitant emotional appeals to protect, defend and take heroic action to restore sacred national space, territory and homelands (Miller‐Idriss, 2020).

The third impact that has emerged as a result of the relationship between nationalism and COVID‐19 is the rise in anti‐government extremism and conspirational sedition (Finkelstein et al., 2020). Anti‐government and apocalyptic far right extremists have rapidly grown in online and offline presence across the United States and Europe, in part through organized protests against state and national shelter in place orders. Calls for violent uprising against the state, political opponents and law enforcement—resulting in part from widely circulating misinformation and disinformation about governments' responses to COVID‐19—have already inspired several violent attacks on law enforcement and at least two planned or enacted plots against hospitals. The growing popularity of conspiracies about a “deep state” and an apparent new convergence among anti‐government groups across the political spectrum—including anti‐vaxxers and flat Earthers, QAnon conspiracy theorists, guns' rights advocates, patriot militias and White supremacist extremists—have created a combustive mix that brings a high risk of serious violence, particularly as we head into a likely second wave of spiking infections and shut downs in the fall of 2020. Conspiracy theories about governments' and corporations' plans to use a vaccine to microchip, neuter or control citizens are also circulating widely in extremist circles, which suggests that nation‐states have a significant implementation challenge ahead of them even after a vaccine is successfully produced.

### 4.2 Will COVID‐19 fuel ethnic and nationalist conflict?

COVID‐19 is likely to fuel ethnic and nationalist conflict in several ways. In the global north, as discussed above, rising xenophobia, conspiracy‐fueled anti‐Asian and anti‐Semitic violence and anti‐immigrant hate are already prevalent during the COVID‐19 pandemic. The potential short‐ and long‐term impacts of school and university closures on youth radicalization are also significant. In the United States alone, over 70 million youth in the primary, secondary and postsecondary systems are currently affected by school and college closures. This has led to massive increases in online engagement in ways that create incalculable risks of engagement with extremist material and recruiters. Shortly after the pandemic began, US federal law enforcement issued warnings about the increased risk of child exploitation as a result of highly online youth presence, combined with reduced parental/caregiver supervision and lessoned interactions with other trusted adult networks, including teachers, coaches, youth group leaders and adult relatives outside the home (Federal Bureau of Investigation, 2020, Online). Similar risks exist for online radicalization (e.g., see State of New Jersey Office of Homeland Security and Preparedness, 2020). The impact of extraordinary amounts of time spent online during the COVID‐19 pandemic—along with a risk in the drivers and grievances that create susceptibility to radicalization, such as anxiety, uncertainty, isolation and lack of purpose—will be clearer as time passes, but should be understood for now as a high‐risk situation related to potential future violent extremism and terror.

In the global south, COVID‐19 will potentially exacerbate ethnic tensions or fuel new ones in already‐fragile states. A heightened lack of trust between local communities and governments or international organizations is part of the problem—in some cases, caused by very real abuses and instances of violence perpetrated by some frontline police and military responders during COVID‐19 curfew enforcement. In places where trust in governments is already low, or where there are existing grievances about inequitable distribution of resources, uneven responses in health care provision or distribution of resources can fuel ethnic conflict. These vertical tensions (between communities and authorities) are matched by deeper horizontal tensions between ethnic groups as shelter‐in‐place orders have reversed gains that had been made through promising communal engagement programmes that brought people together across dividing lines. As families retreat into ethnic communities, the fragile bonds from emerging cross‐ethnic forms of engagement and cooperation are at risk. Finally, both kinds of tensions—vertical and horizontal—are further heightened through the actions of bad actors who have circulated unreliable sources of information, disinformation, misinformation and conspiracy theories about the virus. Some campaigns have targeted ethnic minorities through labels like the “Rohinga virus,” the “Muslim virus” or the “refugee virus,” aiming to produce fear and uncertainty and incite conflict (see Search for Common Ground, n.d.).

### 4.3 Will COVID‐19 reinforce or erode the nation‐state in the long run?

I would expect to see splintering on this question, for several reasons. One has to do with the issues of trust in government discussed above. In countries where the national response has strengthened public trust in the government—such as Germany and New Zealand—the nation‐state will likely be strengthened. But in places where trust is weakened as a result of the government's response to COVID‐19, including in the United States but also in more fragile states in the global south, the nation‐state will likely be further eroded. The widespread circulation of misinformation, disinformation and conspiracy theories related to the virus and a COVID‐19 vaccine will also exacerbate declines in the nation‐state's power, particularly in states where elected officials have failed to counter or have actively supported some conspiracy theories, even prior to COVID‐19 (see Rosenblum & Muirhead, 2019).

The same is likely true for the ways that COVID‐19 has illuminated existing disparities in health care provision across ethnic and racial groups. The drastically different infection and mortality rates for minorities compared with Whites in the United States, for example, make it clearer than ever that the nation‐state does not serve all its people equally. The uneven loss of life for Black Americans and communities of colour should be a wake‐up call to nation‐states and their citizens about the need for systematic change in social services and health care provision, as well as the need to address ongoing legacies of structural racism and discrimination. I would argue that the extent to which nation‐states respond to these grievances will play a big part in whether the pandemic ultimately is a strengthening or a weakening force for the nation‐state more generally.

Finally, increases in ethnic group conflict and political or ethnic group polarization and hate in the wake of COVID‐19 continue will also likely have differential impacts, depending on how states react. Countries whose local, regional and national leadership firmly and unequivocally condemns hate and scapegoating related to the virus may be able to come out of the pandemic with stronger and more resilient communities and nation‐states. But in places where political leaders ignore or exacerbate these tensions and contribute to further polarization, it is hard to see how COVID‐19 will not contribute to the further decline of the nation‐state and the people's identification with it.

## 5 ERIC TAYLOR WOODS (UNIVERSITY OF EAST LONDON) AND ROBERT SCHERTZER (UNIVERSITY OF TORONTO)

We approach all three questions through a lens that conceives of the COVID‐19 pandemic as a crisis. This enables us to construct a typology of the differing ways that crises can impact the development of nationalism. We then use this typology to frame our discussion of how the pandemic could shape nationalism and how nationalism in turn could shape the response to the pandemic.

### 5.1 How should we understand the relationship between nationalism and COVID‐19?

COVID‐19 constitutes a severe global threat. It has significant potential to trigger multiple, cascading *crises* in nearly every aspect of our lives. In addition to the presence of a threat, crises typically involve systemic disruption, uncertainty and stress (Brecher, 2019; Quarantelli & Dynes, 1977; Rosenthal et al., 1989). As a result of this widespread upheaval, crises have a high potential for triggering change (Falleti & Lynch, 2009: 1155). The concept of crisis has not been a specific focus in the field of nationalism studies. That being said, events that could readily be defined as crises, such as warfare, revolution or economic catastrophe, have been central to research on nationalism. This literature suggests that crises can impact the development of nationalism in three distinct ways: they can be (1) constitutive, (2) amplifying, or (3) transformative

*Crises as constitutive events.* Crises, particularly those that are associated with revolution, can be constitutive events in the formation of new nationalisms. Indeed, the French Revolution of 1789 is often depicted as *the* formative event for the worldwide spread of nationalism. During a revolution, the struggle against perceived illegitimate rule can provide a catalyst for the emergence of nationalist sentiment—the idea that “we” constitute our own nation and therefore ought to have political autonomy (see Hobsbawm, 2010; Bell, 2001). Similarly, warfare can be a powerful catalyst for the emergence of nationalist sentiment through conflict with a common threat (Hutchinson, 2017, pp. 36–42).

*Crises as amplifying events.* Crises can also have an amplifying effect on existing nationalisms. As such, they can reinforce both solidarity and division within and between national communities. Solidarity is often expressed through a “rally around the flag effect,” in which people unite under national leaders during the crisis (Brody & Shapiro, 1989; Mueller, 1970; Oneal, Lian, & Joyner, 1996). Nationalism can also provide a collective cipher for succour and inspiration during a crisis, whereby myths, symbols and practices associated with past crises are “rediscovered” and applied to the new crisis (Hutchinson, 2006). The crisis might also give rise to new cultural content and practices, which can further reinforce solidarity (Hutchinson, 2006). However, the inevitable search for responsibility that accompanies a crisis can also amplify divisions with perceived malevolent “others,” both within and outside the national community. Thus, during a crisis, it has been widely observed that attacks against internal minorities tend to surge, while the potential for conflict with external adversaries is heightened.

*Crises as transformative events.* As much as crises can amplify existing nationalisms, they can also be transformative. For example, crises can lead to new configurations of cultural boundaries between who is perceived to belong and who does not. Previously excluded minorities might be incorporated into the national “we” as they make common cause against the threat. For example, the war against a genocidal Germany was an important catalyst for the increased social inclusion of Jews in America (Alexander, 2006: Chapter 19). However, by the same token, minorities that were once included, or at least tolerated, might now be excluded if they become associated with the new threat. Thus, after the terrorist attacks of September 2001, Muslims in the West became the new significant “others” (Byng, 2008; Poynting & Mason, 2006). In the international sphere, perceptions of who is the “friend” and “foe” can also undergo reconfiguration during a crisis. This occurred, for example, in the dramatic reversal of how the West perceived the Soviet Union following the World War II.

So, which of these potential pathways might nationalism take in the wake of COVID‐19? It is too early to tell whether this pandemic will be a constitutive event for the rise of new nationalisms. The same goes for whether it will have a transformative impact. There are signs of a potential “Hamiltonian” moment in Europe with the agreement between Germany and France to pursue a €500bn aid package for the EU, but there are no guarantees that all 27 member states will agree to the proposal, nor whether this will persuade the citizens of those states to relinquish their national identities in favour of a pan‐European identity. Nevertheless, in some instances, there are early signals that COVID‐19 could move the boundary of who belongs and who does not. In the United Kingdom, the importance of ethnic minorities to the nation has been made salient through their increased visibility in the professions on the frontlines of the struggle against the pandemic (Hirsch, 2020). This is also happening in Canada, where leaders are considering making asylum seekers permanent residents to recognize their work in long‐term care facilities (Seidle, 2020). But these are only two examples and it is still early days; in many other cases, we are seeing the opposite happen where migrants are being targeted. We therefore think that among the three pathways we described, the most likely impact of COVID‐19 will be to amplify existing nationalisms.

There are already indications that COVID‐19 is amplifying nationalism across numerous contexts. Most national leaders are enjoying a surge of support. Myths and symbols related to how nations endured past crises, such as warfare, have been rediscovered and repurposed by national leaders in order to inspire their constituents as they confront the pandemic. New collective rituals have also emerged, such as weekly national “clapping” for key workers in the United Kingdom, or the newly founded national days of mourning in Spain. But this amplifying effect has not been entirely solidary. For example, in the United States, there has been a surge in racist attacks against Asian Americans (Tavernise & Oppel, 2020). Meanwhile, as we discuss in our response to Question 2, it is fuelling division in the international sphere.

### 5.2 Will COVID‐19 fuel ethnic and nationalist conflict?

The divisive othering and attribution of responsibility that stem from a crisis can increase the likelihood of intrastate political conflict, but not necessarily lead towards interstate violence.

The splitting of populations into categories of “us” and “them” is central to nationalism. As Fredrik Barth (1969) points out in relation to ethnic identity, it is through contact with “others” that we construct a sense of “our” group. This othering tends to also entail a moralizing process that glorifies “us” and vilifies “them” (Schertzer & Woods, 2020a). And therein lies the rub: at times of crisis, this tendency can propel ethnic and national conflict because it creates logics that rationalize violent or discriminatory practices against perceived malign or corrupted “others.” This is because nationalism provides a cultural roadmap for attributing responsibility for a crisis, in the sense that it is typically the vilified “others” that shoulder the blame.

With COVID‐19, attributing responsibility to an “other” is somewhat indirect, because ultimately, the responsibility lies with a virus rather than human actors. COVID‐19 is an “invisible enemy,” as Donald Trump likes to quip. In this regard, the pandemic is akin to a natural disaster. But even natural disasters typically provoke efforts to attribute responsibility to human actors—to lay *blame* at the feet of an individual, group or institution for failing to act appropriately (Bucher, 1957; Yates, 1998). This process of attributing blame can be highly conflictual. As the conflict takes shape, it tends to align with and amplify existing cleavages (Tilley & Hobolt, 2011). For example, after Hurricane Katrina, an emotive struggle over responsibility ensued that ultimately enflamed a longer running conflict over the place of African Americans in America (Eyerman, 2015).

Similar processes are emerging in relation to COVID‐19. The pandemic is amplifying nationalist sentiment (see Legrain, 2020), which is precipitating a “politics of blame.” This is particularly visible in relations between the United States and China. As Hughes discusses in this Exchange, a relationship that was already strained is now rapidly worsening, as the two countries blame one another for the pandemic. There is a fear that these political disputes may lead towards violent conflict. The vastly simplified argument here is that COVID‐19 creates a series of economic, social and political crises that increases incentives and opportunities for interstate conflict. And when rising nationalism is added to the mix, it increases the probability that leaders will opt for war (see Hutchinson, 2017).

In our view, this account gives too much power to nationalism as the key driver of conflict. We know many of the conditions and logics that drive interstate warfare, and COVID‐19 does not necessarily lead us down these pathways. As others have argued, the pandemic has created significant logistical issues for mass troop mobilization, it has shaken the confidence of states and leaders and there is no necessary link between economic downturns and warfare—recessions are a bad predictor of interstate conflict (Posen, 2020; Walt, 2020). While nationalism can shape decisions and introduce irrationality, it does not *necessarily* have the structuring power to overcome the current barriers to interstate warfare. The view that increasing nationalist sentiment will inevitably lead to violent conflict also oversimplifies nationalism. This logic assumes that nationalism is always dangerous and illiberal, which in our view is an outmoded that builds on a normative distinction between bad (ethnic) and good (civic) forms of identity.

What is more likely is that COVID‐19 will amplify *internal* ethnic divisions within states. The process of othering, the search for blame and the calls to protect our “own” are driving a dynamic whereby foreigners and migrants are being targeted in many states. As Miller‐Idriss details in her contribution, Asians in western countries are suffering racist and violent attacks as perceived stand‐ins, carriers and collaborators of the “silent enemy.” Migrants are facing hostility as potentially dangerous vectors of the virus and threats to the host society. Asylum seekers are being denied entry into many countries or held in dangerous camps where they are at greater risk of contracting COVID‐19. In short, some ethnic divisions *within* states are becoming increasingly salient.

This type of internal ethnic conflict is not directly attributable to the pandemic. Rather, it reflects how the internal dynamics of a national community are shaping the response of leaders and the public at a time of crisis. Political culture matters in how COVID‐19 is shaping nationalism: it is the nation's cultural and political characteristics that are driving the emergent dynamics of conflict. These dynamics are not necessarily marching us down a path towards interstate violence, but they are making existing ethnic divisions within and between nations more salient.

### 5.3 Will COVID‐19 reinforce or threaten the nation state in the long run?

The nation‐state has a privileged position in our political order. The international system is based upon the idea that political communities, called nation‐states, deserve autonomy. The logic of nationalism provides legitimacy to this order: it is because states protect and represent a nation that they have sovereignty (Mayall, 1990).

At first blush, we might expect that a global pandemic would erode the status and autonomy of nation‐states: international collaboration and a pooling of resources are necessary to combat the virus. And yet, nation‐states are leading the response to COVID‐19, while the legitimacy of international organizations like the WHO is being questioned. Given these early trends, and what we know about the endurance of nation‐states, in our view, COVID‐19 will likely reinforce the nation‐state.

The early signals point towards a trend of nation‐states greatly increasing their power in the face of COVID‐19. They are reinforcing borders, curtailing migration, limiting internal population movements, spending vast amounts of money on economic stimulus and increasing surveillance of citizens. Many of these moves have come at the cost of individual liberty and privacy (Economist, 2020). Some of these measures will be relatively short lived, and others will likely be difficult to roll back. Regardless, these patterns recentre the state in our lives. They bring the state back into view as a powerful actor (Skocpol, 1985).

But these developments are about more than simply expanding the administrative capacity of states—they also reinforce the nationalist idea that they represent “nations.” Leaders and publics alike have embraced the rationale that increased state authority and power is necessary to protect the safety and way of life of the *nation.* This rationale is evident in the competition over medical supplies, which is increasingly nationalist in tone (Goodman, Thomas, Wee, & Gettleman, 2020). European Union states have worked against one another by limiting the export of protective equipment to other members in need. The United States has taken actions to limit the export of protective equipment to Canada. Conflicts over the production and distribution of an eventual vaccine are already taking shape. Rather than concerted international collaboration and coordination, we are seeing increasingly protectionist approaches driven by the logic of the nation‐state.

At the same time, the nation‐state is facing threats—from below and from above. There is an argument that sub‐state national movements may use COVID‐19 to push for greater autonomy. This could trigger renewed instability, particularly in multinational states. But the evidence so far suggests the contrary. Many multinational states are seeing a remarkable degree of pan‐national solidarity. Both dynamics are playing out now in the United Kingdom: an early surge in solidarity across the union is increasingly diverging along national lines, with Scotland, Wales and Northern Ireland adopting different approaches to the pandemic. However, even if COVID‐19 does destabilize multinational states through national minority mobilization, this does not completely threaten the idea of the nation‐state. National minorities seeking independence are not working to undermine the international society of nation‐states; they are working to join it (Williams & Schertzer, 2019, p. 680).

COVID‐19 may also threaten the nation‐state from above. The economic and political crises that it will inevitably trigger can create opportunities for powerful nation‐states to extend their influence over less powerful ones. In this regard, the pandemic may enable new forms of imperialism, undermining the status and sovereignty of nation‐states. There are clear parallels supporting this argument: “foreign aid” provided by powerful states and institutions during past crises often belied thinly veiled forms of neo‐colonialism (see Charbonneau, 2008; Fieldhouse, 1999; Langan, 2018). But even a resurgent imperialism may not undermine the *idea* of the nation‐state system. History can serve as a guide. During the “Cold War,” the idea of the sovereign nation‐state was strengthened. Despite the widespread influence of the United States and Soviet Union, the idea that the world was fundamentally composed of sovereign nation‐states did not diminish. Therefore, we tend to agree with Greenfeld that the COVID‐19 pandemic will not diminish the nation‐state system in the long run.

If we are right—if COVID‐19 reinforces the nation‐state—then there are some potential perils. The amplification of power and autonomy of nation‐states, paired with limited checks and accountability, may have long‐lasting effects for privacy, security and democracy. People are rightly fearful that newly emboldened nation‐states may hinder the necessary international collaboration to manage the pandemic. But this fear rests on a false dichotomy: a strengthened nation‐state is not irreconcilable with strong international collaboration—quite the opposite (Schertzer & Woods, 2020b). The security and autonomy afforded by the nation‐state can allow actors to engage in meaningful international collaboration. The establishment and growth of our key international institutions and related norms principally stem from actions taken by states, often following major crises. If this collaboration can be undertaken while being mindful of existing power imbalances—listening to the better angels of our nature—it could both assist in the immediate response to the pandemic and improve international institutions and norms over the longer‐term.
